# Socioeconomic Determinants of the Anthropometric Characteristics and Motor Abilities of Polish Male University Students: A Cross-Sectional Study Conducted in 2000–2018

**DOI:** 10.3390/ijerph17041300

**Published:** 2020-02-18

**Authors:** Robert Podstawski, Piotr Markowski, Dariusz Choszcz, Michał Boraczyński, Piotr Gronek

**Affiliations:** 1 Department of Tourism, Recreation and Ecology, Faculty of Geoengineering, University of Warmia and Mazury in Olsztyn, 10-719 Olsztyn, Poland; 2 Department of Heavy Duty Machines and Research Methodology, Faculty of Technical Sciences, University of Warmia and Mazury in Olsztyn, 10-719 Olsztyn, Poland; piotr.markowski@uwm.edu.pl (P.M.); choszczd@uwm.edu.pl (D.C.); 3 Faculty of Health Sciences, Olsztyn University College, 10-283 Olsztyn, Poland; michal.boraczynski@gmail.com; 4 Department of Dance and Gymnastics, University of Physical Education in Poznan, 61-871 Poznań, Poland; gronek@awf.poznan.pl

**Keywords:** motor performance, somatic traits, socioeconomic indicators, first-year university students

## Abstract

*Aim:* The aim of this study was to determine the relationships between socioeconomic factors, anthropometric characteristics and motor abilities of male university students. *Materials and Methods:* The study was conducted from 2000 to 2018 on 2691 male university students aged 19.98 ± 1.05 years, who were randomly selected from students attending obligatory physical education (PE) classes. The participants’ body mass and height were measured, and students participated in 13 motor ability tests that assessed their speed/agility, flexibility, strength and endurance abilities. Multiple independent samples were compared with the Kruskal–Wallis test or the mean-ranks post-hoc test when significant differences were observed in the participants’ motor abilities. *Results:* Factors such as the place of permanent residence, students’ monthly budget, and mother’s and father’s educational background, significantly (*p* < 0.05) influenced the body mass, BMI and motor abilities of first-year university students. The participants’ motor abilities (speed/agility, flexibility—partly, strength, strength endurance, and endurance) were most frequently and most significantly determined by their monthly budgets, and were least frequently and least significantly determined by their place of permanent residence. *Conclusions:* The students’ body height, BMI and motor abilities generally increased with a rise in population in the place of permanent residence, monthly budget, and the parents’ educational attainment.

## 1. Introduction

The relationships between physical activity (PA), physical fitness (PF) and health have been relatively well established in adults [1]. In the developed world, a sedentary lifestyle has been linked with a reduction in PF levels [2]. In recent years, the determinants of PA and, consequently, PF have been explored by models incorporating not only demographic, psychological and social explanatory variables, but also socioeconomic determinants [3]. Socioeconomic determinants are socioeconomic factors (income, parents’ educational background, size and type of the social environment) that significantly influence human anthropometric characteristics (traits that describe body dimensions), such as height, weight, girth, and body fat composition [4], as well as PF (a set of attributes that are either health- or skill-related. The degree to which people have these attributes can be measured with specific tests) [5].

Socioeconomic factors that play an important role in university students’ PA levels include the parents’ educational background, monthly budget, the place of permanent residence, and the type and location of high school [6]. Considerable differences in PA levels can be expected between countries. Such variations have been reported in Europe [7,8], and they suggest that economic development as well as cultural and geopolitical factors are broadly associated with leisure-time PA. Young adults are generally more physically active in highly developed countries, excluding the Mediterranean Region [9]. According to international research, the percentage of sedentary university students varies across countries, from 23% in Western Europe and the USA, to 30% in Central and Eastern Europe, 39% in the Mediterranean region, 42% in the Asia-Pacific region, and 44% in developing countries [9]. A study of PA levels in 51 countries with mostly low or middle incomes revealed several trends [10].

A systematic review of the literature conducted by Wartburton et al. [11] revealed a clear dose–response relationship between PA and selected health indicators, including PF. However, PA and PF exert independent effects on health indicators [12]. The existing research suggests that improvements in PF are most conducive to minimizing selected health risks [13] and that PF exerts a greater influence on health indicators than PA [14]. The above findings suggest that low levels of PF are a risk factor that is directly associated with a sedentary lifestyle [12]. The types of PA performed by university students induce various changes in body composition and motor abilities [15]. Motor abilities are theoretical constructs describing individual traits that are conditioned by bodily structure, energy processes and motor control, and which directly determine the effective performance of specific physical tasks [16]. Longitudinal studies on somatic development have revealed a continued increase in body mass and height as well as varied changes in specific motor abilities in recent decades [17,18,19,20,21,22]. Analyses of the relationship between socioeconomic determinants, anthropometric characteristics and motor abilities provide valuable insights in many practical settings, in particular, in evaluations of PA levels. Individual differences in motor performance are explained by the interaction effects of environmental and biological factors [23].

From a public health perspective, first-year university students have become an important research group in studies conducted around the world. The beginning of university education and the time spent at university are a critical moment in the promotion of a healthy lifestyle. Epidemiological evidence suggests that PA levels decline steadily between high school and college, and university students are not sufficiently active to remain healthy and fit [24]. Research also indicates that the incidence of overweight and obesity is highest between the ages of 18 and 29 [25]. University students are more likely to gain weight than youths who are not enrolled in college [26]. A recent meta-analysis revealed that first-year university students gain approximately 4 lbs (1.81 kg) during a period of 3 to 12 months [27]. In most cases, excess weight contributes to an increase in fat mass [28], and first-year university students tend to maintain the gained weight [29] or even continue to gain weight in successive years of college [30]. University students generally gain weight due to low levels of PA as well as excessive caloric intake. Only 38% of college students participate in regular vigorous activity [31]. A decrease in PA levels was also reported in nearly 50% of college students after graduation [32]. Studies that measure students’ anthropometric characteristics and motor abilities provide valuable inputs and support the implementation of the most effective solutions. However, changes in the somatic and motor development of first-year university students have been insufficiently investigated by cross-sectional and longitudinal studies which are conducted only in selected research centers (tertiary institutions) [33].

In view of the above, the aim of this study was to evaluate the relationships between the anthropometric characteristics and motor abilities of male university students and selected socioeconomic factors, such as mother’s and father’s educational background, monthly budget, and the place of permanent residence.

## 2. Materials and Methods

### 2.1. Participants

The study was conducted from 2000 to 2018 on 2691 full-time, first-year male university students aged 19 ÷ 25 (19.98 ± 1.05 years) who were randomly selected from the students attending obligatory physical education (PE) classes at the University of Warmia and Mazury (UWM) in Olsztyn, Poland. Beginning from 2000, the study was carried out at two-year intervals during the summer semester (April/May). Students were selected randomly on a volunteer basis, and those who wished to participate signed an informed consent form. If the chosen student did not wish to participate in the study, another potential candidate was randomly drawn. Only those students who were absent, for whatever reason, on the day the tests and measurements were performed, were excluded from the study. The participants were selected from among volunteers who did not take any medication or nutritional supplements, were in good health, had no history of blood diseases or diseases affecting biochemical and biomechanical factors. Research has revealed significant gender-related differences in personal, social and environmental factors that influence PA levels [34]. Therefore, we assumed that male and female participants should be tested separately. In this study, only the results scored by men were presented in view of diverse factors or determinants. 

### 2.2. Ethics

The research was carried out upon the prior consent of the Ethical Committee of the UWM in Olsztyn (No. 39/2011). The study involved male student volunteers who signed a written statement of informed consent.

### 2.3. Instruments and Procedures

Body mass (to the nearest 0.1 kg) and body height (to the nearest 0.1 mm) were measured using a calibrated medical scale with a stadiometer (WB-150 ZPU Tryb Wag, Zamość, Poland), and the results were used to calculate the participants’ BMI. Student volunteers participated in thirteen motor ability tests assessing their speed/agility abilities: 4 × 10 m shuttle run (s), 8-s skipping with hand clapping (SHC) test (number of claps), zig-zag run (s); flexibility abilities: standing forward bend (cm), barbell overhead trunk rotation (cm); strength abilities: standing broad jump (cm), sit-ups in 30 s (number of sit-ups), medicine ball (4 kg) forward throw (cm), medicine ball (4 kg) backward throw (cm), pull-ups on bar (number of pulls); strength endurance: 1-min and 3-min Burpee tests (1-MBT, 3-MBT) (number of cycles); and endurance abilities: 12-min Cooper test on a rowing ergometer (m). The reliability of the repeated motor tests was considered high (ICC—intraclass correlation coefficient: 0.84–0.92; CV—coefficient of variation: 1.5%–3.4%). These motor tests had been widely used to analyze the motor abilities of different age groups, including as separate trials to assess specific motor abilities and as part of batteries of tests to evaluate general motor fitness [35,36,37,38]. The validity and reliability of the applied tests were confirmed in research studies [39,40,41,42,43,44,45] In each group, motor ability tests were conducted in the same order, beginning from strength, speed, agility, strength endurance and endurance tests, and ending in flexibility tests. The instructions for each test were given during the PE class, and students were allowed sufficient time to practice. The participants performed the same standard active warm-up exercises for 10 min before each test [46]. 

The participants provided information about their gender, age, parents’ educational background, the place of permanent residence, and monthly budget by filling out a questionnaire. The results of the questionnaire were used to evaluate the impact of selected environmental factors on the students’ performance in motor ability tests.

### 2.4. Statistical Analysis

The results were processed with the use of descriptive statistics (mean, standard deviation, etc.). The results were processed statistically in Statistica PL v. 13 at a significance level of α = 0.05. The null hypothesis postulated that selected socioeconomic factors (mother’s and father’s educational background, monthly budget, and the place of permanent residence) do not influence performance in motor ability tests. The assumptions of the parametric test were not met, i.e., data did not have normal distribution, the study groups had unequal size, and sample variances were not homogeneous; therefore, the Kruskal–Wallis test for comparing many independent samples was used as the non-parametric equivalent of one-way ANOVA. The mean-rank post-hoc test for multiple comparisons was used when significant differences were observed in the participants’ motor abilities.

## 3. Results

The socioeconomic characteristics of the tested subjects are presented in Table 1. The correlations between anthropometric characteristics vs. the place of permanent residence and the students’ monthly budgets were presented in Table 2, and the relationships between motor abilities and remaining environmental factors are presented in Table 3 and Table 4.

### 3.1. Analysis 1: Socioeconomic Characteristics of the Study Group 

More than half of the studied subjects (57.7%) resided permanently in cities with a population of up to 50,000 (P1). Somewhat fewer students (but also more than 50%) were raised by mothers and fathers with secondary education (M2—54.9% and F2—50.9%). A similar percentage of the evaluated subjects had monthly budgets below PLN 1500 (R1—41.0%) and budgets between PLN 1500 and PLN 3000 (R2—42.4%). The smallest proportion of the analyzed students resided in cities with a population above 50,000 (P3—14.3%) and had monthly budgets higher than PLN 3000 (R3—16.6%) (Table 1).

### 3.2. Analysis 2: Socioeconomic vs. Anthropometric Characteristics 

The place of permanent residence, the students’ monthly budget, as well as mother’s and fathers’ educational background had no significant (*p* > 0.05) effect on body mass or test scores. Students residing in cities with a population below and above 50,000 (P2) and students with monthly budgets higher than PLN 1500 (R2, R3), whose mothers and fathers had secondary or university background, were significantly taller than rural dwellers (P1) with monthly budgets below PLN 1500 (R1) and mothers and fathers with primary/vocational education (M1, F1). In addition, students raised by parents with university education were taller than their counterparts whose parents had secondary school education. The inhabitants of rural areas and cities with a population below 50,000 (P1, P2), with monthly budgets below PLN 1500 (R1), whose mothers and fathers had primary/vocational educational background (M1, F1), had significantly higher BMI (*p* < 0.05) than the residents of cities with a population higher than 50,000 (P3), subjects with monthly budgets above PLN 1500 (R2, R3), as well as subjects whose parents had secondary and university educational background (M2, M3 and F2, F3, respectively). Significant differences in favor of students with better educated parents were also noted between M2 > M3 and F2 > F3. The average BMI values were within the norm in all groups (Table 2).

### 3.3. Analysis 3: Place of Permanent Residence and Students’ Monthly Budget vs. Motor Abilities

The place of permanent residence and the students’ monthly budget had no significant (*p* > 0.05) effect on the scores in the 4 × 10 m shuttle run, barbell overhead trunk rotation, 30-s sit-ups, and the 12-min rowing ergometer test. The place of permanent residence also did not induce significant differences in the results of the 8-s SHC, zig-zag run, downward bend, pull-ups on a bar, or 1- and 3-MBTs. The scores in the medicine ball forward throw were not significantly (*p* > 0.05) correlated with the students’ monthly budgets. The residents of cities with a population below and above 50,000 (P2, P3) performed significantly better in the standing broad jump and the medicine ball backward throw than their rural counterparts (P1). In the medicine ball forward-throw, the above relationship was noted only in the residents of cities with a population higher than 50,000 (P3). Students with a monthly budget higher than PLN 1500 (R2, R3) scored significantly (*p* < 0.05) higher in the 8-s SHC, zig-zag run, downward bend (only R3), medicine ball backward-throw and 1- and 3-MBT than those with a monthly budget below PLN 1500 (R1). In the above tests, significant differences were also found between (R3) > (R2). Differences were also noted between (R1) < (R2) in the standing broad jump, and between (R1) < (R3) in the number of pull-ups (Table 3).

### 3.4. Analysis 4: Mother’s and Father’s Educational Background vs. Motor Abilities 

Students raised by better educated mothers and fathers also scored higher in the 8-s SHC, 4 × 10 m shuttle run, and the standing broad jump. The mother’s educational background was also correlated with the scores in the 12-min rowing ergometer test, and the father’s educational background—with the scores in the zig-zag run, barbell overhead trunk rotation, 30-s sit-ups, and medicine ball backward throw (*p* < 0.05). Mother’s educational attainment did not significantly (*p* > 0.05) affect the scores in the remaining motor ability tests: the zig-zag run, downward bend, barbell overhead trunk rotation 30-s sit-ups, medicine ball backward throw, pull-ups on a bar, and 1- and 3- MBTs. In turn, father’s educational attainment had no significant effect in the following motor ability tests: downward bend, medicine ball forward throw, pull-ups on a bar, 1- and 3-MBT, and the 12-min rowing ergometer test (Table 3).

## 4. Discussion

Considerable research has been done on academic youths who are an important social group from the public health perspective [47]. In the present study, an attempt was made to evaluate the relationships between selected socioeconomic factors (mother’s and father’s educational background, students’ monthly budget, and the place of permanent residence) vs. the anthropometric characteristics and motor abilities of university students. These factors were tested on the assumption that PA levels cannot be explained or predicted by a single variable, and that variation in PA levels should be analyzed based on the interaction effects of several factors [34,48]. According to Hendry et al. [49], PA levels are directly related to the social models of reference and the reinforcement or support received for such practices. Therefore, individual PA levels are considerably influenced by the support received from significant others [8,50]. Family and friends are thus among the main socialization agents of PA. In the current study, mother’s and father’s educational background significantly influenced the students’ body height and BMI. Father’s educational attainment significantly affected the level of motor abilities in seven fitness tests, and mother’s educational background exerted a significant influence on the scores in five tests; therefore, it was assumed that father’s attitude towards PA had a more profound influence on the students’ PF than mother’s attitude. The above influence was most significantly expressed by the participants’ scores in speed/agility tests (8-s SHC, 4 × 10 m shuttle run, zig-ag run), strength tests (standing broad jump, 30-s sit-ups, medicine ball backward-throw), and, partly, in flexibility tests (barbell overhead trunk rotation).

The highest number (10 cases) of correlations and the strongest correlations were noted between the students’ motor abilities and their monthly budget. Similarly to other socioeconomic factors, the monthly budget significantly (*p* < 0.001 in most cases) determined the students’ body height, BMI and strength abilities (standing broad jump, medicine ball backward-throw, pull-ups on a bar), strength endurance (1- and 3-MBT), speed/ability (8-s SHC, zig-zag run) and sagittal spinal flexibility (downward bend from standing position). Zhang et al. [51] examined the influence of socioeconomic determinants on the PF of children (<18 years of age), which is a predictor of adult health and disease. A study performed in the USA demonstrated that children from lower-income families tended to have lower PF scores [52]. A study of adolescents from nine European countries revealed that higher SES is strongly associated with improved PF measured by cardiorespiratory fitness (CF) and muscular strength [53]. In a cross-sectional study, Sandercock et al. [54] investigated the association between SES and the PF of 52,187 ninth-grade students in Colombia, a rapidly developing upper-middle income country. They found that area-level SES was positively associated with both upper- and lower-body muscular fitness, whereas CF was not affected by SES. When the results were adjusted for family and area-level SES, students attending private schools and those residing in urban areas had better muscular strength and CF. Family income was not associated with any of the PF measures after adjustment for area-level SES. Family income could be a more distal determinant of the area of residence and type of school, i.e., factors that exert a more direct effect on PF. Even if the effects of family income are not significant when area-level SES and type of school are controlled for, family income could still be implicated in the causal chain between SES and PF indicators. The above implies that an increase in income could enable families to move to better areas and access schools that offer more opportunities for improving PF. The exact number of university students who work part-time is difficult to determine. Several studies demonstrated that 50%–60% of all university students are engaged in some form of part-time employment, but the vast majority of students continue to receive financial support from their parents [55]. The study by Sandercock et al. [54] contradicted the results reported by Otero et al., and it demonstrated that Colombian students with higher area-level SES had lower handgrip strength (the same measure was used by Sandercock et al. to determine upper-body muscular fitness). The association between SES and PF measures is also influenced by age [56]. Otero et al. examined children aged 8–17 years, whereas Sandercock et al. [54] evaluated only ninth graders aged 14–16 years. The above discrepancies could also be attributed to differences in methodology, including sampling design, the applied measures and the analytical strategy.

Similarly to other socioeconomic factors, the place of permanent residence significantly affected the students’ body height and BMI, and it significantly determined their strength abilities (standing broad jump, medicine ball backward- and forward-throws). Parents’ educational background and the place of residence should not be regarded as factors that directly influence PF, but as parameters indicating that individual development is conditioned by various socioeconomic factors [57]. Research has revealed considerable differences in the PF of Polish youths from different socioeconomic backgrounds, and it demonstrated that PF reflects on the stratification of Polish society [58,59]. Uppal and Sareen [60] found that rural students were characterized by higher cardiovascular fitness than their peers residing in urban areas. A comparative study of PF components revealed that rural female students scored higher in strength, endurance, speed and agility tests, whereas students residing in urban areas were heavier and more flexible [1]. Rural youths are characterized by a relatively higher level of strength abilities than their urban peers [61]. Adolescents living in lower-SES areas often have limited access to recreational facilities, which is associated with lower PA levels [62]. For this reason, public health programs should aim to improve the built environment in poorer neighborhoods to provide more opportunities for physical activity and reduce health disparities [54]. Research also demonstrated that PA programs initiated in schools contribute to higher PA levels [63]. The above implies that schools with a higher proportion of students residing in areas with low SES, in particular, public schools, should analyze local needs and opportunities and implement programs that promote physical activity among students.

The identified variations in the PF of university students from different socioeconomic backgrounds resemble the results of a study conducted in the 1980s, where social differences in somatic development were determined based on body height [64]. University students raised by parents with a university education and residing in large cities are generally characterized by the highest PF levels [65,66,67].

The PA and PF levels of university students tend to be more influenced by cultural factors than by SES; therefore, any attempts to stimulate PF in this population should focus on improving the students’ motivation, promoting awareness toward a healthy lifestyle and the relevant hierarchy of values. Despite the fact that university students do not attach great importance to PF, programs that focus on the promotion of PF as a significant value and an essential lifestyle component should be incorporated into general educational curricula [68].

## 5. Strengths and Limitations of the Study

In the last decade, very few studies have investigated the relationships between environmental factors and the motor abilities of university students, and research involving first-year university students is even more scant. The vast majority of studies exploring the physical fitness of university students were conducted in the 1970s, 1980s and 1990s. The present study was conducted on a relatively large, representative and homogeneous sample, and the results constitute valuable material which can be compared with the findings of previous and, possibly, future research. The only limitation of this research stems from the fact that the study was conducted only at the UWM in Olsztyn, mainly due to organizational problems. Studies involving a large and representative sample and a high number of motor tests are very difficult to perform due to time and logistic limitations (base). However, the UWM in Olsztyn is a public university which admits students from all Polish regions.

## 6. Conclusions

Socioeconomic factors such as the place of permanent residence, students’ monthly budget and mother’s and father’s educational background, significantly influence the body height, BMI and motor abilities of first-year university students. Students residing in large cities, students with a higher monthly budget, and students raised by better educated parents, are taller and score higher in motor ability tests. Students’ motor abilities (speed/agility, strength, strength endurance and, partly, flexibility) were most frequently and most significantly determined by their monthly budgets, and were least frequently and least significantly determined by their place of permanent residence.

## Figures and Tables

**Table 1:** The socioeconomic characteristics of the evaluated university students.

Characteristics	Categories	Group	N	%
**Place of permanent residence**	Village	P1	754	28.0
	City < 50,000	P2	1552	57.7
	City > 50,000	P3	385	14.3
**Students’ monthly budget**	< PLN 1500	R1	1103	41.0
	PLN 1501 to 3000	R2	1142	42.4
	> PLN 3000	R3	446	16.6
**Mother’s educational background**	Primary/Vocational	M1	534	19.8
	Secondary	M2	1478	54.9
	University	M3	679	25.2
**Father’s educational background**	Primary/Vocational	F1	643	23.9
	Secondary	F2	1371	50.9
	University	F3	677	25.2

Note: P1—village; P2—city < 50,000; P3—city > 50,000; R1—< PLN 1500; R2—from PLN 1501 to 3000; R3—> PLN 3000, M1—M1—primary school/vocational school, M2—secondary school, M3—university, F1–F3: Father’s educational background (identical criteria as in mother’s educational background).

**Table 2:** Place of residence and monthly budget, mother’s and father’s educational background vs. anthropometric characteristics.

Indicators		Place of Residence					Monthly Budget				
		Group	Mean ± SD	N	*p* Value	Significant Differences	Group	Mean ± SD	N	*p* Value	Significant Differences
**Anthropometric characteristics**	**Body mass (kg)**	P1	76.7 ±9.60	754	0.418	-	R1	77.3 ± 24.06	1103	0.670	-
		P2	77.3 ± 21.01	1552			R2	76.9 ± 9.65	1142		
		P3	76.2 ± 9.17	385			R3	76.5 ± 9.73	446		
	**Body height (cm)**	P1	180.2 ± 5.67	754	0.000	P1 < P2, P3	R1	180.3 ± 5.60	1103	0.000	R1 < R2, R3
		P2	181.3 ± 6.09	1552			R2	181.6 ± 6.31	1142		
		P3	182.1 ± 6.56	385			R3	182.0 ± 6.35	446		
	**BMI (kg/m^2^)**	P1	23.7 ± 2.85	754	0.000	P1, P2> P3	R1	23.8 ± 7.43	1103	0.000	R1 > R2, R3
		P2	23.5 ± 6.42	1552			R2	23.3 ± 2.71	1142		
		P3	23.0 ± 2.54	385			R3	23.1 ± 2.71	446		
		**Mother’s educational background**					**Father’s educational background**				
	**Body mass (kg)**	M1	77.8 ± 10.14	534	0.060	-	F1	78.1 ± 30.44	643	0.485	-
		M2	76.9 ± 21.28	1478			F2	76.6 ± 9.73	1371		
		M3	76.6 ± 9.59	679			F3	76.6 ± 9.49	677		
	**Body height (cm)**	M1	180.1 ± 5.46	534	0.000	M1 < M2, M3; M2 < M3	F1	179.7 ± 5.84	643	0.000	F1 < F2, F3; F2 < F3
		M2	181.0 ± 6.03	1478			F2	181.2 ± 5.97	1371		
		M3	182.3 ± 6.44	679			F3	182.4 ± 6.23	677		
	**BMI (kg/m^2^)**	M1	24.0 ± 2.87	534	0.000	M1 > M2, M3; M2 > M3	F1	24.2 ± 9.45	643	0.000	F1 > F2, F3; F2 > F3
		M2	23.5 ± 6.53	1478			F2	23.4 ± 2.72	1371		
		M3	23.1 ± 2.75	679			F3	23.0 ± 2.66	677		

Note: P1—village; P2—city < 50,000; P3—city > 50,000; R1— < PLN 1500; R2—from PLN 1501 to 3000; R3—> PLN 3000. M1—primary school/vocational school; M2—secondary school; M3—university; F1–F3: Father’s educational background (identical criteria as in mother’s educational background). BMI: body mass index. SD: standard deviation.

**Table 3:** Place of residence and students’ monthly budget vs. motor indicators.

Indicators		Place of Residence					Monthly Budget				
		Group	Mean ± SD	N	*p* Value	Significant Differences	Group	Mean ± SD	N	*p* Value	Significant Differences
**Speed/agility**	**8-s SHC (number of claps)**	P1	24.0 ± 3.36	754	0.271	-	R1	23.8 ± 3.35	1103	0.001	R1 < R2, R3
		P2	24.2 ± 3.34	1552			R2	24.3 ± 3.33	1142		
		P3	24.1 ± 3.36	385			R3	24.4 ± 3.35	446		
	4 × 10 m shuttle run * (s)	P1	10.8 ± 1.02	754	0.507	-	R1	10.8 ± 1.12	1103	0.267	-
		P2	10.8 ± 1.13	1552			R2	10.8 ± 1.08	1142		
		P3	10.8 ± 1.10	385			R3	10.8 ± 1.09	446		
	Zig-zag run * (cm)	P1	25.3 ± 2.58	522	0.798	-	R1	26.3 ± 2.60	706	0.000	R1 < R2, R3; R2 < R3
		P2	25.2 ± 2.77	1156			R2	24.9 ± 2.65	802		
		P3	25.2 ± 2.94	177			R3	23.9 ± 2.33	347		
**Flexibility**	**Downward bend (cm)**	P1	6.6 ± 6.06	754	0.811	-	R1	6.6 ± 5.85	1103	0.001	R1 < R3; R2 < R3
		P2	6.9 ± 6.41	1552			R2	6.7 ± 6.35	1142		
		P3	6.9 ± 6.15	385			R3	7.6 ± 7.01	446		
	Barbell * overhead trunk rotation (cm)	P1	78.12 ± 12.71	522	0.521	-	R1	79.0 ± 13.86	706	0.823	-
		P2	79.1 ± 14.39	1156			R2	78.7 ± 13.73	802		
		P3	78.0 ± 12.79	177			R3	78.4 ± 13.79	347		
**Strength**	**Standing broad jump (cm)**	P1	210.6 ± 22.21	754	0.001	P1 < P2, P3	R1	211.6 ± 22.49	1103	0.020	R1 < R2
		P2	213.5 ± 23.10	1552			R2	213.8 ± 22.61	1142		
		P3	214.8 ± 21.53	385			R3	213.6 ± 23.16	446		
	**30-s sit-ups (number of sit-ups)**	P1	23.6 ± 3.67	754	0.138	-	R1	23.7 ± 3.64	1103	0.881	-
		P2	23.6 ± 3.72	1552			R2	23.7 ± 3.53	1142		
		P3	24.0 ± 3.61	385			R3	23.4 ± 4.21	446		
	**Medicine ball** **backward throw (cm)**	P1	1005.4 ± 181.68	754	0.005	P1 < P2, P3	R1	979.6 ± 173.57	1103	0.000	R1 < R2, R3; R2 < R3
		P2	1024.7 ± 193.21	1552			R2	1029.5 ± 190.21	1142		
		P3	1039.1 ± 193.37	385			R3	1103.8 ± 200.73	446		
	**Medicine ball forward throw (cm)**	P1	867.8 ± 170.61	522	0.044	P1 < P3	R1	865.7 ± 173.53	706	0.073	-
		P2	875.7 ± 170.54	1156			R2	878.4 ± 173.40	802		
		P3	901.8 ± 180.92	177			R3	891.4 ± 163.02	347		
	**Pull-ups (number of pull-ups)**	P1	4.8 ± 3.89	754	0.790	-	R1	4.7 ± 3.74	1103	0.005	R1 < R3
		P2	4.8 ± 3.79	1552			R2	4.8 ± 3.77	1142		
		P3	4.9 ± 3.66	385			R3	5.2 ± 4.04	446		
**Strength** **endurance**	**1-MBT (number of cycles)**	P1	23.4 ± 3.49	754	0.611	-	R1	22.5 ± 3.20	1103	0.000	R1 < R2, R3; R2 < R3
		P2	23.5 ± 3.54	1552			R2	23.9 ± 3.45	1142		
		P3	23.4 ± 3.64	385			R3	24.7 ± 3.96	446		
	**3-MBT (number of cycles)**	P1	57.6 ± 10.28	522	0.949	-	R1	55.6 ± 9.20	706	0.000	R1 < R2, R3; R2 < R3
		P2	57.4 ± 10.74	1156			R2	57.9 ± 10.82	802		
		P3	57.6 ± 9.83	177			R3	60.36 ± 11.55	347		
**Endurance**	**12-min rowing ergometer test (m)**	P1	2548.8 ± 313.06	522	0.467	-	R1	2547.4 ± 310.29	706	0.513	-
		P2	2523.9 ± 328.45	1156			R2	2522.7 ± 330.00	802		
		P3	2525.4 ± 323.20	177			R3	2517.2 ± 335.12	347		

Note: *—a lower number denotes a better score; therefore, the plus/minus sign was reversed for significant differences. P1— village; P2— city < 50,000; P3—city > 50,000; R1—< PLN 1500; R2—from PLN 1501 to 3000; R3— > PLN 3000. SHC: skipping and hand clapping. MBT: minute Burpee tests.

**Table 4:** Parents’ educational background vs. motor indicators.

Indicators		Mother’s Educational Background					Father’s Educational Background				
		Group	Mean ± SD	N	*p* Value	Significant Differences	Group	Mean ± SD	N	*p* Value	Significant Differences
**Speed/agility**	**8-s SHC (number of claps)**	M1	23.7 ± 3.24	534	0.015	M1 < M2, M3	F1	22.9 ± 3.07	643	0.000	F1 < F2, F3; F2 < F3
		M2	24.2 ± 3.38	1478			F2	24.0 ± 3.20	1371		
		M3	24.2 ± 3.35	679			F3	25.4 ± 3.44	677		
	4 × 10 m shuttle * run (s)	M1	10.9 ± 1.07	534	0.008	M1 < M2	F1	11.3 ± 1.03	643	0.000	F1 < F2, F3; F2 < F3
		M2	10.7 ± 1.06	1478			F2	10.8 ± 1.10	1371		
		M3	10.8 ± 1.19	679			F3	10.2 ± 0.85	677		
	Zig-zag run * (cm)	M1	25.4 ± 2.71	347	0.398	-	F1	25.4 ± 2.77	423	0.030	F1 < F3
		M2	25.2 ± 2.71	1047			F2	25.3 ± 2.67	965		
		M3	25.2 ± 2.81	461			F3	25.0 ± 2.83	467		
**Flexibility**	**Downward bend (cm)**	M1	6.6 ± 6.22	534	0.426	-	F1	7.0 ± 6.45	643	0.680	-
		M2	6.8 ± 6.24	1478			F2	6.7 ± 6.32	1371		
		M3	7.0 ± 6.40	679			F3	6.9 ± 6.02	677		
	Barbell overhead trunk rotation * (cm)	M1	79.4 ± 14.44	347	0.120	-	F1	82.5 ± 13.43	423	0.000	F1 < F2, F3; F2 < F3
		M2	78.9 ± 13.42	1047			F2	78.9 ± 13.92	965		
		M3	77.8 ± 14.09	461			F3	74.9 ± 12.83	467		
**Strength**	**Standing broad jump (cm)**	M1	200.5 ± 20.70	534	0.000	M1 < M2, M3; M2 < M3	F1	210.4 ± 22.71	643	0.000	F1 < F2,F3
		M2	212.4 ± 21.00	1478			F2	213.0 ± 22.74	1371		
		M3	223.6 ± 22.48	679			F3	215.0 ± 22.29	677		
	**30-s sit-ups** **(number of sit-ups)**	M1	23.3 ± 3.73	534	0.081	-	F1	22.4 ± 3.30	643	0.000	F1 < F2, F3; F2 < F3
		M2	23.7 ± 3.68	1478			F2	23.6 ± 3.59	1371		
		M3	23.7 ± 3.69	679			F3	24.8 ± 3.91	677		
	**Medicine ball** **backward throw (cm)**	M1	1009.4 ± 186.77	534	0.142	-	F1	1011.0 ± 190.48	643	0.001	F1, F2 < F3
		M2	1021.4 ± 190.88	1478			F2	1014.7 ± 189.17	1371		
		M3	1030.8 ± 191.65	679			F3	1044.7 ± 190.86	677		
	**Medicine ball forward throw (cm)**	M1	836.9 ± 145.66	347	0.000	M1 < M2, M3; M2 < M3	F1	874.2 ± 162.60	423	0.486	-
		M2	876.9 ± 164.74	1047			F2	873.4 ± 173.91	965		
		M3	903.3 ± 198.16	461			F3	882.8 ± 175.34	467		
	**Pull-ups (number of pull-ups)**	M1	4.9 ± 4.01	534	0.368	-	F1	4.8 ± 3.77	643	0.054	-
		M2	4.9 ± 3.77	1478			F2	4.7 ± 3.83	1371		
		M3	4.7 ± 3.71	679			F3	5.1 ± 3.77	677		
**Strength** **endurance**	**1-MBT (number of cycles)**	M1	23.5 ± 3.44	534	0.950	-	F1	23.3 ± 3.55	643	0.090	-
		M2	23.5 ± 3.56	1478			F2	23.5 ± 3.53	1371		
		M3	23.5 ± 3.58	679			F3	23.7 ± 3.54	677		
	**3-MBT (number of cycles)**	M1	57.3 ± 11.17	347	0.531	-	F1	56.9 ± 11.08	423	0.264	-
		M2	57.5 ± 10.52	1047			F2	57.4 ± 10.40	965		
		M3	57.5 ± 10.03	461			F3	58.0 ± 10.24	467		
**Endurance**	**12-min rowing ergometer test** **(m)**	M1	2368.9 ± 295.28	347	0.000	M1 < M2, M3; M2 < M3	F1	2501.8 ± 336.51	423	0.096	-
		M2	2530.9 ± 312.52	1047			F2	2535.4 ± 319.7	965		
		M3	2653.5 ± 315.91	461			F3	2548.5 ± 319.01	467		

Note: *—a lower number denotes a better score; therefore, the plus/minus sign was reversed for significant differences. M1—primary school/vocational school, M2—secondary school, M3—university, F1–F3: Father’s educational background (identical criteria as in mother’s educational background).
